# Designed Surface Topographies Control ICAM-1 Expression in Tonsil-Derived Human Stromal Cells

**DOI:** 10.3389/fbioe.2018.00087

**Published:** 2018-06-28

**Authors:** Aliaksei S. Vasilevich, Frédéric Mourcin, Anouk Mentink, Frits Hulshof, Nick Beijer, Yiping Zhao, Marloes Levers, Bernke Papenburg, Shantanu Singh, Anne E. Carpenter, Dimitrios Stamatialis, Clemens van Blitterswijk, Karin Tarte, Jan de Boer

**Affiliations:** ^1^Laboratory for Cell Biology-Inspired Tissue Engineering, MERLN Institute for Technology-Inspired Regenerative Medicine, Maastricht University, Maastricht, Netherlands; ^2^Institut National de la Santé et de la Recherche Médicale, U917, Equipe Labelisée Ligue Contre le Cancer, Université Rennes, I'Etablissement Français du Sang Bretagne, Rennes, France; ^3^Department of Biomaterials Science and Technology, MIRA Institute for Biomedical Technology and Technical Medicine, University of Twente, Enschede, Netherlands; ^4^Materiomics BV, Maastricht, Netherlands; ^5^Imaging Platform, Broad Institute of MIT and Harvard, Cambridge, MA, United States; ^6^Department of Complex Tissue Regeneration, MERLN Institute for Technology-Inspired Regenerative Medicine, Maastricht University, Maastricht, Netherlands

**Keywords:** mechanobiology, surface topography, fibroblastic reticular cells, lymph node, ICAM-1

## Abstract

Fibroblastic reticular cells (FRCs), the T-cell zone stromal cell subtype in the lymph nodes, create a scaffold for adhesion and migration of immune cells, thus allowing them to communicate. Although known to be important for the initiation of immune responses, studies about FRCs and their interactions have been impeded because FRCs are limited in availability and lose their function upon culture expansion. To circumvent these limitations, stromal cell precursors can be mechanotranduced to form mature FRCs. Here, we used a library of designed surface topographies to trigger FRC differentiation from tonsil-derived stromal cells (TSCs). Undifferentiated TSCs were seeded on a TopoChip containing 2176 different topographies in culture medium without differentiation factors, then monitored cell morphology and the levels of ICAM-1, a marker of FRC differentiation. We identified 112 and 72 surfaces that upregulated and downregulated, respectively, ICAM-1 expression. By monitoring cell morphology, and expression of the FRC differentiation marker ICAM-1 via image analysis and machine learning, we discovered correlations between ICAM-1 expression, cell shape and design of surface topographies and confirmed our findings by using flow cytometry. Our findings confirmed that TSCs are mechano-responsive cells and identified particular topographies that can be used to improve FRC differentiation protocols.

## Introduction

Successful engineering of an artificial lymph node would enable *in vitro* investigation of the immune system, allow toxicological tests on a system closely mimicking the *in vivo* situation, and, ultimately, clinical transplantation (Cupedo et al., 2012). The lymph nodes are secondary lymphoid organs that control the immune system: they maintain hematopoietic cell functioning by serving as a tissue scaffold and provide pro-survival signals. They also facilitate the formation of antigen-presenting sites, which promotes the immune response to antigens. Lymph nodes consist of hematopoietic and non-hematopoietic cells that are closely interconnected. Moreover, they harbor unique microenvironments, where either T cells or B cells are located and become activated (Crivellato et al., 2004; Cupedo et al., 2012). Stromal cells of lymph nodes are difficult to purify and culture due to their scarcity (< 1% in secondary lymphoid organs (SLOs), strong interaction with extracellular matrix compounds (Fletcher et al., 2011), and rapid loss of functionality when removed from their native environment (Zeng et al., 2011). The culture of primary lymph node stromal cells has been successfully accomplished by only few groups (Katakai et al., 2004; Fletcher et al., 2011; Onder et al., 2012). The most abundant stromal cell type in lymph nodes is the fibroblastic reticular cell (FRC), which builds a three-dimensional network. (Katakai et al., 2004; Link et al., 2007). One of their key roles is to secrete cytokines such as CCL19/21 that specifically attract naïve T, naïve B, and mature dendritic cells, and they further act as a scaffold for anchoring and navigating cells, allowing them to interact and initiate an immune response (Turley et al., 2010; Malhotra et al., 2013).

An alternative to studying primary FRCs is to induce FRC differentiation from mesenchymal progenitor cells, derived from tonsil. We and others have shown that human SLOs contain bona-fide mesenchymal stromal cells (MSCs) that can be robustly differentiated to FRC in response to a combination of tumor necrosis factor-α (TNF-α) and lymphotoxin-α1β2 (LT-α1β2), the two main factors involved in differentiation and maintenance of SLO (Ame-Thomas et al., 2007; Fletcher et al., 2015; Bar-Ephraim et al., 2016). We reported that exposure of tonsil-derived stromal cells (TSCs, a polyclonal cell type that can be cultured from fresh tonsil tissue) to Tumor Necrosis Factor-α (TNF-α) and Lymphotoxin-α1β2 (LT-α1β2) leads to expression of FRC specific markers *in vitro*. These markers include adhesion molecules

CXCL12 chemokines (Ame-Thomas et al., 2007). Moreover, it was shown that ICAM-1 together with VCAM-1 expression reflects the differentiation process from stromal progenitors to FRC (Bénézech et al., 2012). Human tonsils can be obtained after a routine tonsillectomy. Tonsil stromal cells can be isolated by digestion of tonsils in collagenase and DNAse, centrifugation on a discontinuous Percoll gradient and collection of plastic- adherent cells. During expansion, cells lose their phenotype and thus it is not possible to track back their anatomical location and function in the tonsil, however, it was shown that these cells express mesenchymal markers CD90, CD73, CD105 and CXCL12 and exhibit a spindle-shaped fibroblastic morphology (Ame-Thomas et al., 2007). Moreover, the bone marrow and the adipose tissue can be considerate as alternative sources of stromal cells. BM-MSCs (Bone Marrow Mesenchymal Stromal Cells) and ASCs (Adipose Stromal Cells) are mostly used for regenerative medicine (Gentile et al., 2012) or for their immunosuppressive properties (Uccelli et al., 2008). While MSCs derived from bone marrow (BM-MSCs) can also be used for FRC differentiation, these cells are more sensitive to replicative senescence (Tarte et al., 2010) that reduces their immunological properties (Loisel et al., 2017). Adipose precursors from adipose tissue can differentiate into a variety of lymph node stromal cells (Bénézech et al., 2012). We demonstrated that adipose stromal cells (ASCs), like BM-MSCs, were able to acquire a FRC-like phenotype after TNF-α/LT-α1β2 stimulation (Pandey et al., 2017).

All three stromal cell sources—TSCs, ASCs and BM-MSCs—can be routinely obtained and used for achieving the FRC-like phenotype. However, it is known that the anatomical source of stromal cells can predefine their differentiation capabilities: for example, BM-MSCs overall yield better osteogenesis compared to ASCs (Liao and Chen, 2014), which in turn have better adipogenic differentiation ability compared to BM-MSCs *in vitro* (Tsuji et al., 2014). Taking this into account, we have chosen TSCs as the source for FRC differentiation for these studies. Cytokine-induced FRC differentiation can be complemented with biomaterials manipulation. And potentially allows simultaneous co-culturing of multiple cell types which all required their own differentiation factors and may not be compatible with each other. It has been widely recognized that material properties such as chemistry, stiffness, and surface topography affect the biological processes of cells that grow on them (Guvendiren and Burdick, 2013; Luong-Van et al., 2013; Janson and Putnam, 2015). Interestingly, recent studies show that interstitial flow causes mechano-induced FRC differentiation of cancer-associated fibroblasts and subsequent modulation of T cell differentiation (Swartz and Lund, 2012). This finding suggests that mechanotransduction can be used as a tool to induce FRC differentiation.

One of the approaches to induce mechanical loading on cells is to introduce microtopography on the surface on which the cells are growing. Topographical cues have been repeatedly shown to dramatically influence cell behavior and phenotype (Unadkat et al., 2011; Kolind et al., 2012; Wong et al., 2014). For instance, we discovered surfaces with topographical structures were able to maintain Oct4 expression, support proliferation and cell-cell adhesion of human-induced pluripotent stem cells without added growth factors (Reimer et al., 2016). Furthermore, we have demonstrated that surface topographies exert a mitogenic effect on BM-MSCs (Unadkat et al., 2011) and have profound effects on cell shapes (Hulsman et al., 2015). Recently, we have shown that some topographies increase mineralization in BM-MSCs and improve bone bonding *in vivo* (Hulshof et al., 2017). The advantages of this approach are that topographies can be designed *in silico* and produced on virtually any surface, from culture plates to medical devices, using micro-fabrication technologies (Zhao et al., 2013; Ryan et al., 2015; Sackmann et al., 2015).

To study systematically the effect of topography on TSCs differentiation, we employed the TopoChip, a microtopography screening platform developed in our laboratory (Unadkat et al., 2011). The TopoChip enables the assessment of cell response to 2176 unique topographies in a single high-throughput slide. Topographical features were randomly selected from the *in silico* library of more than 150 million topographies, which were designed using an algorithm that generates patterns based on three simple geometric elements – circles, triangles, and rectangles (Supplementary Figure 1).

We cultured human TSCs on the TopoChip platform, and evaluated, by high-content imaging, cellular response, cell morphology and the expression of the phenotypic marker ICAM-1. We identify topographical patterns that modulate ICAM-1 expression in TSCs, complementing differentiation into FRC by chemical inducers of differentiation.

## Materials and methods

### Cell culture

Stromal cells were obtained from human tonsils collected from children undergoing routine tonsillectomy, after informed consent as described previously (Ame-Thomas et al., 2007). Cells were cultured in αMEM media supplemented with 10% FBS at 37°C in a humid atmosphere with 5% CO^2^ unless stated differently. For induction of FRC phenotype, cells were treated with TNF-α (10 ng/mL) and LT-α1β2 (100 ng/mL; R&D Systems, Abingdon, United Kingdom). On TopoChips, cells were seeded at a density of 10,000 cells/cm^2^, using a seeding device (Unadkat et al., 2013) and cultured for 48 h (Supplementary Figure 2).

#### Adipogenesis

To induce adipogenesis, cells were cultured for 3 weeks in adipogenic media (DMEM (Gibco, 41-965-062), 100 U/ml penicillin +100 mg/ml streptomycin (Gibco, 15140-122), 10% fetal bovine serum, 0.2 mM Indomethacin (Sigma, 57413), 0.5 mM IBMX (Sigma, I5879), 10^−6^ M dexamethasone (Sigma, D8893), 10 ug/ml insulin (human, Sigma, I9278). Cells were seeded at a density of 15,000 cells/cm^2^; media was refreshed twice per week. To visualize lipid formation, cells were stained with Oil Red O as described before (De Boer et al., 2004). Briefly, cells were fixed with 10% formalin for 30 min at room temperature, rinsed with a water and washed with 60 % isopropanol. The sample was stained for 5 min in freshly filtered Oil Red O solution (stock: 500 mg Oil Red O (Sigma, O0625), 99 ml isopropanol, 1 ml water; stain: 15 ml stock + 10 ml water).

Oil Red O staining was quantified by extraction with 1 ml of 4% Igepal (Sigma, 56741) in isopropanol for 15 min by shaking at room temperature. Absorbance was measured at 540 nm.

#### Mineralization

To induce mineralization, cells were cultured for 4 weeks in mineralization media (α-MEM (Gibco, 22-571-038), 10% fetal bovine serum (Sigma), 2 mM L-glutamine (Gibco, 25030), 0.2 mM ascorbic acid (Sigma, A8960), 100 U/ml penicillin + 100 mg/ml streptomycin (Gibco, 15140-122), 10^−8^ M dexamethasone (Sigma, D8893) plus 0.01 M β-glycerol phosphate (Sigma, 50020). Media was changed twice per week. To visualize mineral formation, samples were stained with Alizarin Red. Cells were fixed with neutral buffered formalin (10%) for 30 min, rinsed with water, and Alizarin Red was added for 45 min. Calcium deposition was quantified using a Calcium Assay Kit (Gibco, 10010-056) as described in the manual.

### Staining and imaging

Cells were fixed with 4% paraformaldehyde for 10 min; afterwards, samples were permeabilized with 0.5% Tween-20 for 20 min with following blocking step in 5% bovine serum albumin (BSA; Sigma) at room temperature for 30 min. Samples were incubated with primary antibodies against ICAM-1 (Abcam, ab53013) overnight at 4°C. Labeling with secondary antibodies conjugated to fluorochrome Alexa 594 and phalloidin conjugated to fluorochrome Alexa 488 was performed for 1 h at room temperature, followed by 10-min incubation with DAPI. Imaging was acquired on a Hamamatsu Nanozoomer Slide Scanner II.

### Image and data analysis

Open source software Cell Profiler (CP) was used for the image analysis (Carpenter et al., 2006). In order to perform automated image analysis in CP, a robust pipeline able to recognize different cell features was built. Data analysis was performed using R, a programming language and software environment for statistical computing and graphics. Potential miss segmentation of cells was detected based on cell area and perimeter. Cells with cell perimeter and area that exceeded 1.5 quantiles from the median among all replicas for a particular surface were excluded from further analysis.

ICAM-1–positive or –negative cells were identified based on a threshold value. The threshold value was determined as the intersection of ICAM-1 cells median intensities distributions of positive and negative controls (Figure **4B**) and corresponded to the 93rd percentile of all ICAM-1 median cell intensities in the negative control (Figure **4B**). The threshold value was determined for each TopoChip replica separately and equalled the 93rd percentile of all ICAM-1 median intensities per replica. This adaptive threshold strategy allowed us to take a variation of median intensities between TopoChip replicas into account (Supplementary Figure 5).

To evaluate the correlation between ICAM-1 expression and surface design parameters, we trained a machine-learning model with 10-fold cross-validation with 75% of the data by employing a random forest classification algorithm. We analyzed design parameters of 72 low-scoring units and 112 high-scoring units that had a significant difference in frequency of ICAM-1–positive cells. The accuracy of the obtained model was assessed on a held-out data set that was not used for model training (25% of the data). To ensure that the model was robust, we performed model training 100 times with random splitting of the data in training and testing sets. Models were trained with 10-fold cross-validation in the “caret” package (Kuhn, 2008).

### Flow cytometry

BM-MSCs and TSCs were characterized by flow cytometry as described previously (Mentink et al., 2013). Briefly, after trypsinization, cells were incubated for 30 min in blocking buffer (5% BSA (Sigma) in PBS), followed by incubation for 30 min with primary antibodies (1:50 dilution) or with an isotype control antibody. Cells were washed 3 times and incubated with secondary antibodies (1:100 dilution) for 30 min, followed by 2 times washing. To characterize surface markers, we used the following antibodies CD105, CD90, CD11b, CD19, CD45 (R&D Systems), and CD34 (Abcam). Expression levels were analyzed on a FACS Calibur (Becton Dickinson Immunocytometry Systems, Mountain View, CA).

For the validation on enlarged surfaces, cells were stained using the following monoclonal antibodies (mAbs): CD54 PE (Clone 84H10, Beckman Coulter) and Podoplanin/gp38 PerCP-efluor710 (clone NZ-1.3, Affymetrix). Appropriate isotype-matched mAbs were used as negative controls. Cells were analyzed using a Gallios (Beckman Coulter) flow cytometer and Kaluza 1.2 software (Beckman Coulter).

### Quantitative RT-PCR

RNA was extracted using the Nucleospin RNA XS kit (Macherey-Nagel) and cDNA was generated using Superscript II reverse transcriptase and random hexamers (Life Technologies). For quantitative RT-PCR, assay-on-demand primers and probes, and TaqMan Universal Master Mix from Applied Biosystems (Life Technologies) were used. Gene expression was measured using StepOnePlus (Life Technologies) based on the ΔC_t_ calculation method. PUM1 was used as an internal standard gene. For each sample, the *C*_*t*_ value for the gene of interest was determined, normalized to its respective value for PUM1, and compared to the value obtained with a flat surface.

### Topochip and fabrication of enlarged surfaces

The TopoChip was designed by selecting 2176 algorithm-generated topographies from an *in-silico* library and fabricated with the unique topographies in a 66 × 66 array of 300 × 300 μm wells, each known as a TopoUnit. Each 2 × 2 cm TopoChip includes duplicates of all 2176 unique topographies as well as 4 unpatterned TopoUnits (Unadkat et al., 2011).

Both the TopoChips and enlarged surface areas of hit topographies were prepared by hot embossing of (poly)styrene (PS) films (Goodfellow) (Zhao et al., 2013). Briefly, standard photolithography and deep reactive etching were used to produce the inverse structures of the topographies on a silicon wafer. The silicon master mold was then used to make a positive mold in poly(dimethylsiloxane) (PDMS). Subsequently, a second negative mold in OrmoStamp hybrid polymer (micro resist technology Gmbh) was obtained from the PDMS mold. This mold serves as the template for hot embossing (10 bar at 140°C for 5 min) the TopoChips and enlarged topographically enhanced surface areas in 190-μm-thick PS films. Prior to cell culture, the topographically enhanced PS films were O_2_-plasma treated to increase protein attachment to the substrate surface in order to increase cell attachment.

### Statistical analyses ad data visualization

Statistical analysis was performed in R ver. 3.2.5, graphs were generated in R package ggplot2 (Wickham, 2009) or in Graph Pad version 6.0. Unless stated differently, statistical analysis was performed with Student *t*-test, with a significance threshold of *p* < 0.05.

## Results

### Characterization of TSCs

**Figure 1:**
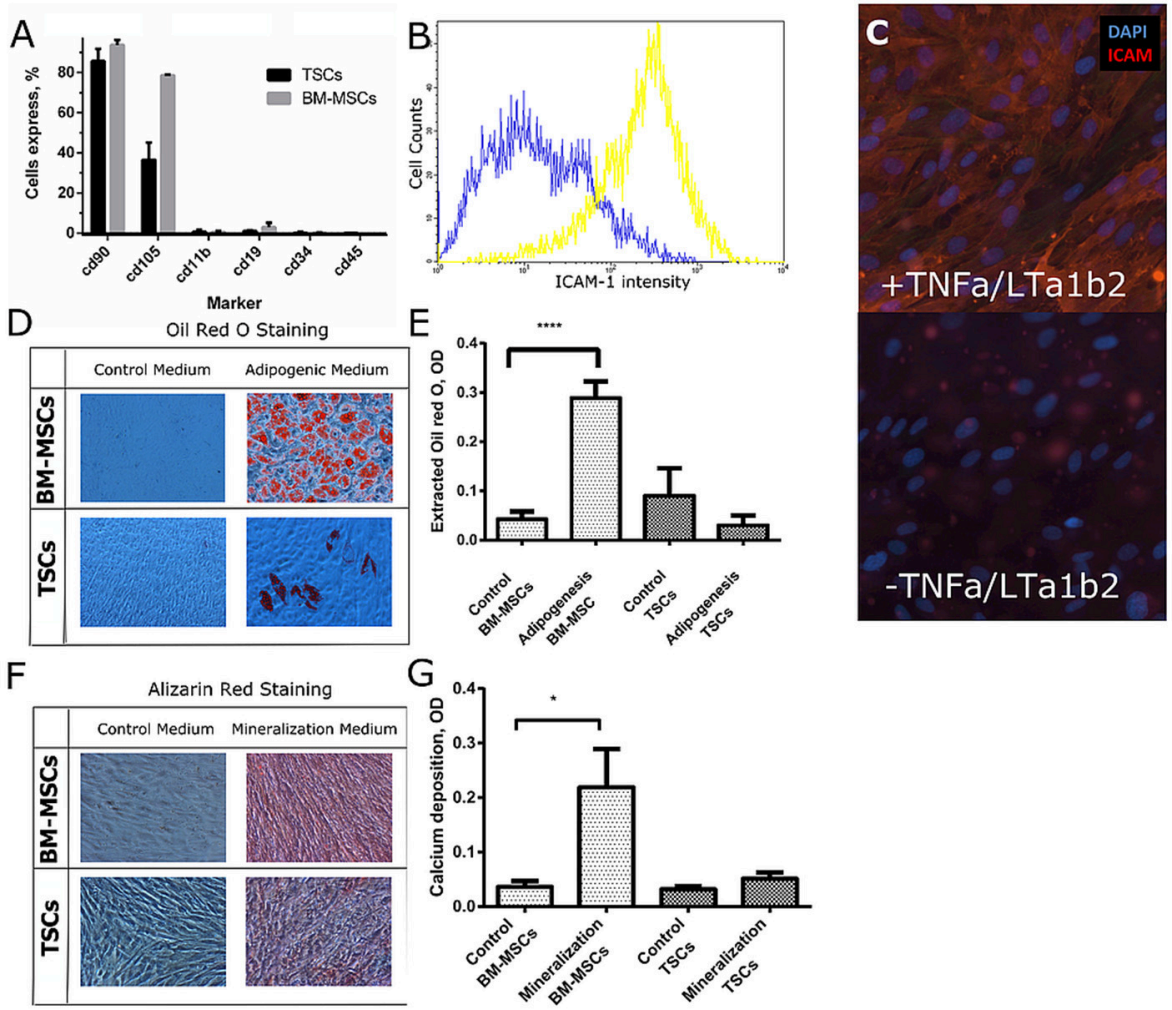
Characterization of TSCs in comparison with BM-MSCs. **(A)** BM-MSCs and TSCs were screened on a panel of mesenchymal markers. **(B,C)** TSCs were cultured with and without TNF-α and LT-α1β2 for 48 h. ICAM-1 expression was assessed by FACS analysis **(B)**, and by immunofluorescent staining **(C)**. **(D)** BM-MSCs and TSCs were cultured in adipogenic medium for 3 weeks; lipids were stained with oil red O. **(E)** Lipid quantification was performed by lysis of stained lipids with Oil red O in a mixture of isopropanol and Igepal. **(F)** BM-MSCs and TSCs were cultured in osteogenic medium for 4 weeks; deposited calcium was stained with Alizarin red. **(G)** Calcium deposition was quantified using a calcium assay kit. *Indicates significant difference with a *p* < 0.05. ****Indicates significant difference with a *p* < 0.0001.

We isolated TSCs from a human tonsil and characterized surface marker expression and their response to a panel of differentiation media. BM-MSCs were used as a reference cell type (Figure 1A). Flow cytometry indicated that 37% of TSCs, compared to 79% of BM-MSCs, expressed the CD105 mesenchymal marker (Ame-Thomas et al., 2007), and the mesenchymal marker CD90 was highly expressed in both TSCs and BM-MSCs. Neither TSCs nor BM-MSCs expressed markers indicative of macrophages (CD11b), B-cells (CD19), hematopoietic stem cells (CD34), or leukocytes (CD45).

To assess if TSCs cells can differentiate into FRCs, we cultured them for 48 h in media containing TNF-α and LT-α1β2. We used ICAM-1 as a phenotypic marker associated with FRC differentiation, which was previously shown to correlate with induction of FRC-like phenotype in TSCs (Ame-Thomas et al., 2007). We observed upregulation of ICAM-1 by flow cytometry (Figure 1B). Moreover, we were able to capture the difference in ICAM-1 expression clearly by immunofluorescence imaging (Figure 1C), which is essential for image-based screening assays (Bray and Carpenter, 2004).

To investigate multipotency of TSCs, we exposed them to adipogenic media and mineralization media. Following exposure to adipogenic media for 3 weeks, we observed most BM-MSCs with abundant lipid droplet formation (Figure 1D) but only few TSCs with lipids (Figures 1D,E). Upon exposure to mineralization medium for 4 weeks, mineralization was clearly observed in BM-MSCs but only occasionally detected minerals in TSCs (Figure 1F). As expected, the amount of deposited calcium in BM-MSCs treated with mineralization media was higher than in the untreated condition, while calcium was not significantly increased in TSCs (Figure 1G). These results demonstrate that the osteogenic and adipogenic differentiation capacity of TSCs was low compared to that of BM-MSCs.

### TSCs respond to surface topography

**Figure 2:**
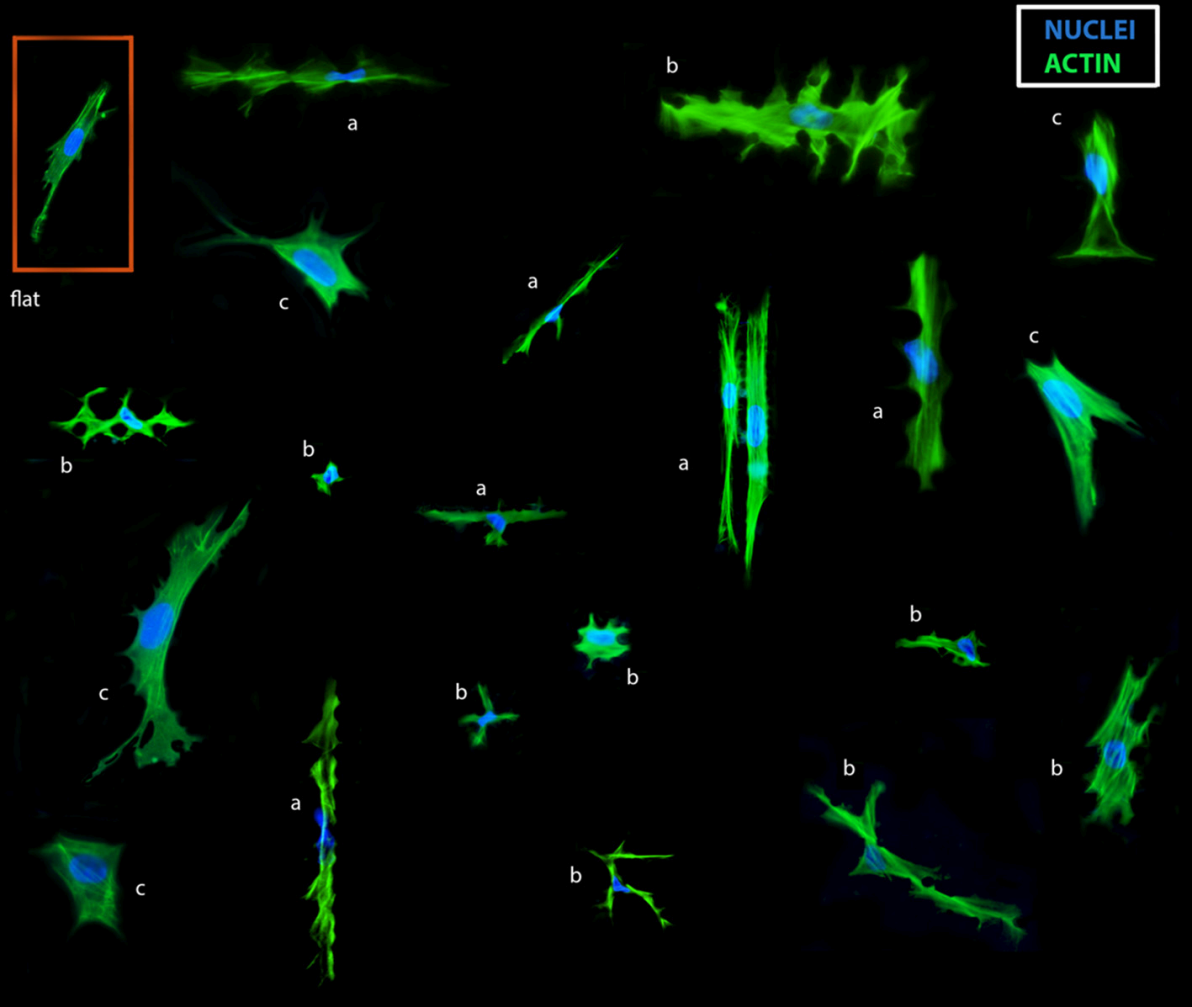
Collage of TSC shapes on the TopoChip. TSCs were cultured for 48 h on a TopoChip in basic media and images of cells on different surfaces are displayed. Actin (green) was stained with phalloidin, DNA (blue) was stained with DAPI: cells in-between pillars: **(a)** extremely elongated cells, **(b)** cells with multiple lobes. Cells on top of pillars: **(c)** large cells.

**Figure 3:**
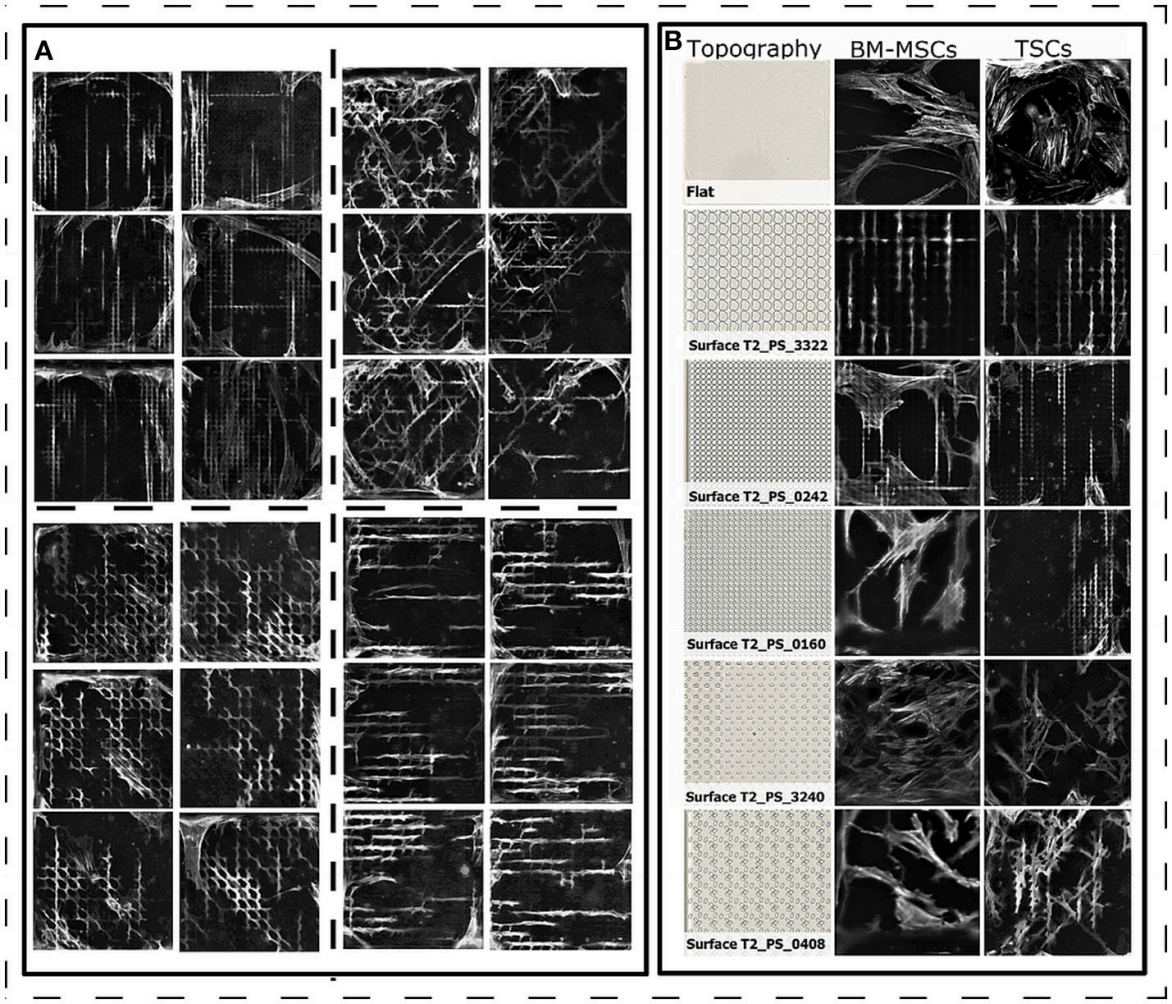
Cell morphology of TSCs on polystyrene surfaces. **(A)** Diverse cell shapes on the TopoChip, that are highly reproducible within replicas (close located images). Actin staining is in gray. TSCs cells were cultured for 2 days in basic media. **(B)** Comparison of BM-MSC and TSC shapes on topographies with a different design. Actin staining is in gray. BM-MSCs were cultured in basic media for 5 days on titanium-coated topographies. TSCs cells were cultured for 2 days in basic media on polystyrene topographies.

To evaluate the response of TSCs to surface topography, we seeded cells on polystyrene TopoChips, and cultured them for 48 h in basic medium and stained them with phalloidin and DAPI to visualize the cytoskeleton and nuclei, respectively. On flat control surfaces, TSCs displayed a typical spindle shape and oval nuclei (Figure 2), but showed very diverse cell shapes on the different topographies (Figure 2). For example, some topographies induced an extremely elongated morphology, cells with multiple lobes bulging out of the main body, or cells with extremely deformed nuclei. Within units and between replicas of the same topographies, cell shape was highly reproducible (Figure 3A). We noticed that cell shape was affected by the spacing between topography pillars. Previously, we were able to construct a computational model that was able to predict cell morphology based on surface design (Hulsman et al., 2015).

Next, we compared cell morphologies of TSCs and BM-MSCs grown on the same topographies. Images of BM-MSCs were taken from a previous experiment, where cells were cultured on titanium-coated surfaces (Hulshof et al., 2017). Here, we noted that the morphology of TSCs and BM-MSCs were similar on some topographies (for example, surface T2_PS_3322, Figure 3B) but different for others. For instance, on surface T2_PS_0242, TSCs grew in the valleys between the topographical pillars, whereas many BM-MSCs grew on top of the pillars. This may be due to the difference in cell size or cytoskeletal organizations (Figure 3B). In addition, observed changes can be due to the different protein absorption on the two materials (Stevens and George, 2005). However, we have compared MSC morphologies on titanium and polystyrene flat surfaces (Supplementary Figure 6) and found out that cells morphology is on flat surfaces is comparable, regardless of the chemistry. In order to draw strong conclusions, a pairwise comparison of BM-MSC and TSC morphologies on the TopoChip is required though, which is beyond the aim of the current paper.

### Surface topographies induce differences in ICAM-1 expression

**Figure 4:**
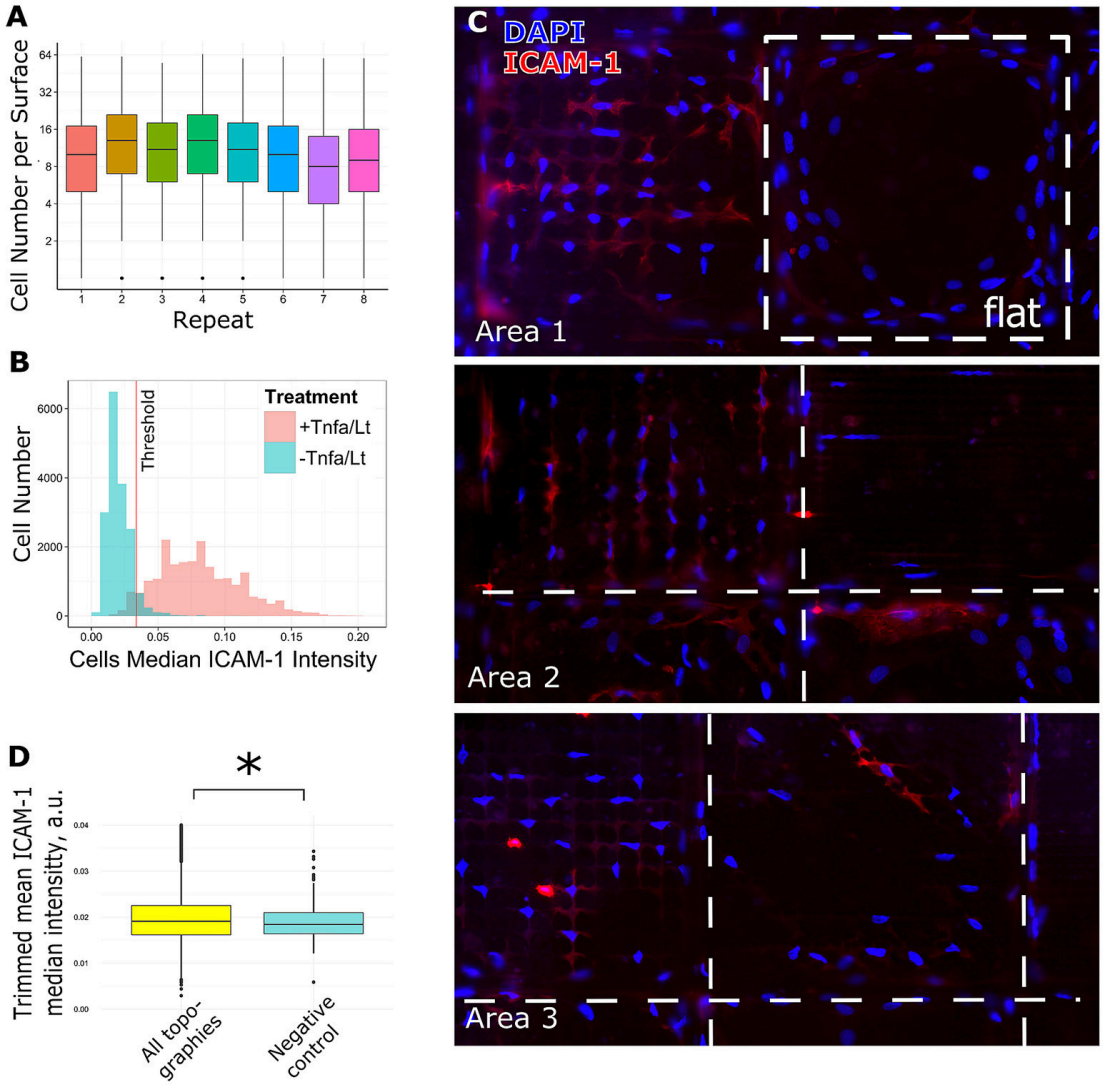
Analysis of screening data and selection of hits. TSCs cells were cultured on 8 Topochips in basic media for 48 h. **(A)** Distribution of cell number per TopoUnit among all replicas. Cell numbers were counted by DAPI staining. **(B)** TSCs cells were cultured with or without TNF-α and LT-α1β2 on flat polystyrene for 48 h. The difference in ICAM-1 expression in samples treated and untreated with TNF-α and LT-α1β2., positive and negative control correspondingly. The threshold for identification of ICAM-1 positive vs. negative cells is shown as a red line. **(C)** The difference in ICAM-1 expression in cells, shown on random areas of the Topochip. DNA (blue) was stained with DAPI, ICAM-1 (red) was stained using an antibody against ICAM-1. Dashed white line shows borders between different topographies. **(D)** Median expression of ICAM-1 in cells on topographies was higher than in negative control and significantly different with a *p*-value less than 0.01 using a Wilcoxon rank sum test. *indicates significant difference with a *p*-value < 0.01.

To assess the effect of surface topography on ICAM-1 expression, we cultured TSCs on eight TopoChips for 48 h in basic medium without TNF-α and LT-α1β2. Each TopoChip contains 2176 unique topographies, in duplicate, resulting in 16 replicas per unique topography. As positive and negative controls, respectively, we cultured TSCs on flat polystyrene ofapproximately equal area to the TopoChip with or without TNF-α and LT-α1β2. Cells were stained with DAPI to identify nuclei and phalloidin to visualize the cytoskeleton. The median cell number per unique topography varied between TopoChip replicas, with a range of 8–13 cells that corresponds to ~160 cells per surface (Figure 4A). Next, we visually observed ICAM-1 staining as a marker of FRC differentiation using a robust image analysis pipeline using the open-source software, CellProfiler (Kamentsky et al., 2011). We found an increase in ICAM-1 staining in TSCs cultured on the TopoChips (Figure 4B), with the striking differences in ICAM-1 expression between topographies (Figure 4C). ICAM-1 expression was higher on the majority of topographies compared to the unpatterned surfaces in the TopoChips (Figure 4C). Quantitative analysis of the images showed that the median ICAM-1 expression on all topographies was significantly higher than that found from the negative control, which is TSCs cultured on flat polystyrene in basic media (Figure 4D).

**Figure 5:**
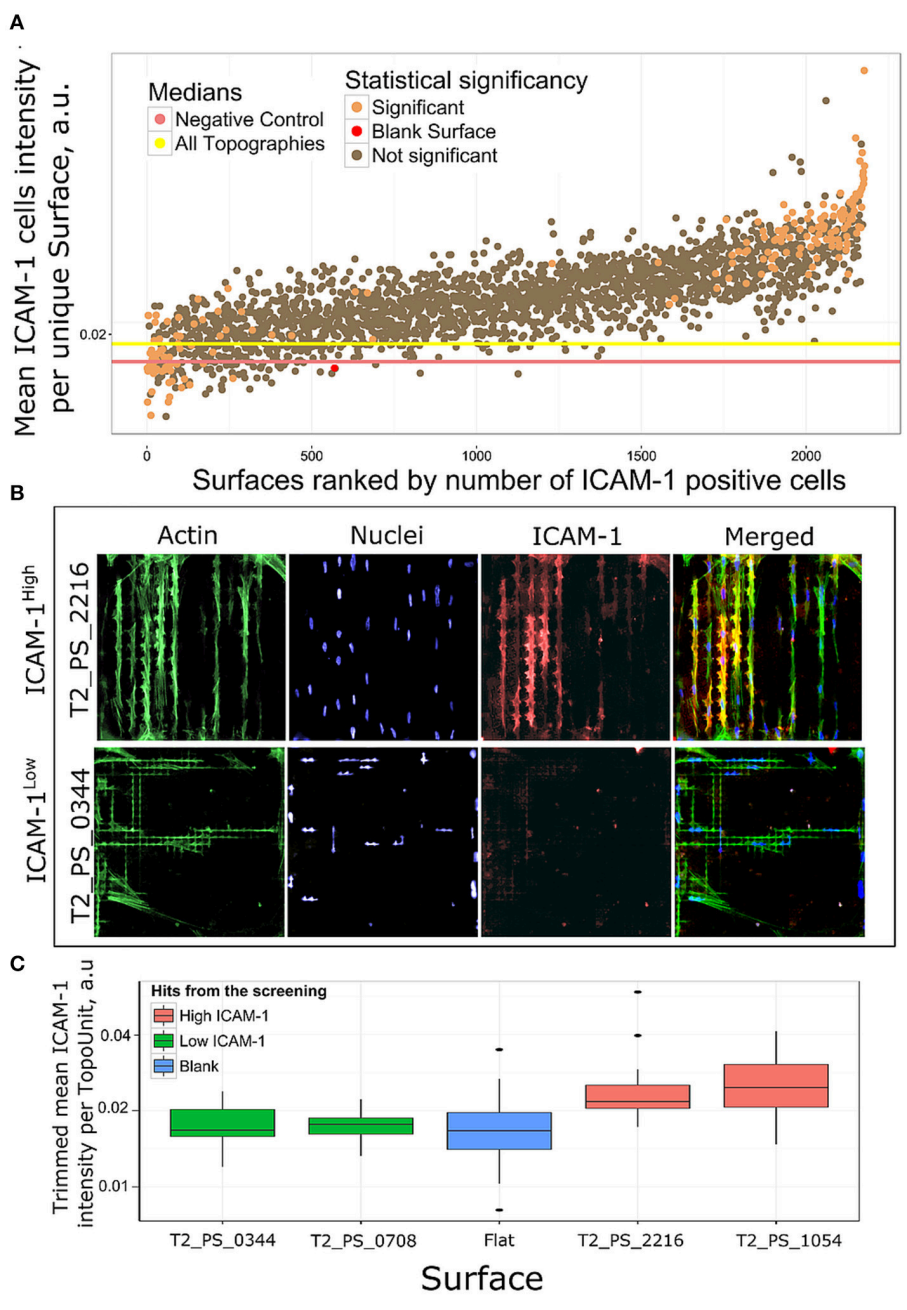
Rank of surfaces based on ICAM-1 intensity. **(A)** Mean ICAM-1 expression in all replicas was ranked by a number of ICAM-1 positive cells. Using chi-square test we identified 184 surfaces with significantly different number of ICAM-1 positive cells in comparison with flat polystyrene, orange dots. **(B)** Immunofluorescent images of cell morphologies represented by actin (green), nuclei (blue) and FRC differentiation marker ICAM-1 (red) staining on a high scoring surface T2_PS_2216 and low scoring T2_PS_0344. Actin was stained with phalloidin, nuclei were stained with DAPI. **(C)** Distribution of ICAM-1 expression in surfaces selected for further validation.

To determine how different topographies affect the expression of ICAM-1, we ranked surfaces based on the ratio of ICAM-1–positive cells in all replicas per unique surface. Cells were classified as either ICAM-1–positive or –negative based on a threshold value of fluorescence intensity (Figure 4B). Among the ICAM-1–positive hits, we identified ICAM-1^High^ and ICAM-1^Low^ surfaces by employing a chi-square test: we identified 112 surfaces that had a significantly higher number of ICAM-1–positive cells compared to the negative control (ICAM-1^High^ surfaces) and 72 with a lower number of ICAM-1–positive cells (ICAM-1^Low^) (Figure 5A). The morphology of TSCs from ICAM-1^High^ and ICAM-1^Low^ surfaces were different (Figure 5B). Among the hits, we noticed that ICAM-1^High^ and ICAM-1^Low^ surfaces not only have a drastically different level of ICAM-1 expression but also unique cell shapes (Figure 5B), which will be discussed below. From the hits, we selected four topographies that belong to either ICAM-1^High^ or ICAM-1^Low^ (Figure 5C, Supplementary Figure 3) and have different cell shapes (Supplementary Figure 4). These topographies were used for further validation studies. We discovered that topographies have a diverse effect on ICAM-1 expression in TSCs. TSCs on flat topographies are among the lowest ICAM-1 expression from all the tested surfaces. We were able to rank topographies based on ICAM-1 presence and selected topographies for validation studies taking into account cells shapes. Selected surfaces had a very clear contrasting expression of ICAM-1 which was visible on immunofluorescent images and was validated by the analysis (Figure 5B, Supplementary Figure 3).

### ICAM-1 expression correlates to surface design parameters and cell shape

**Figure 6:**
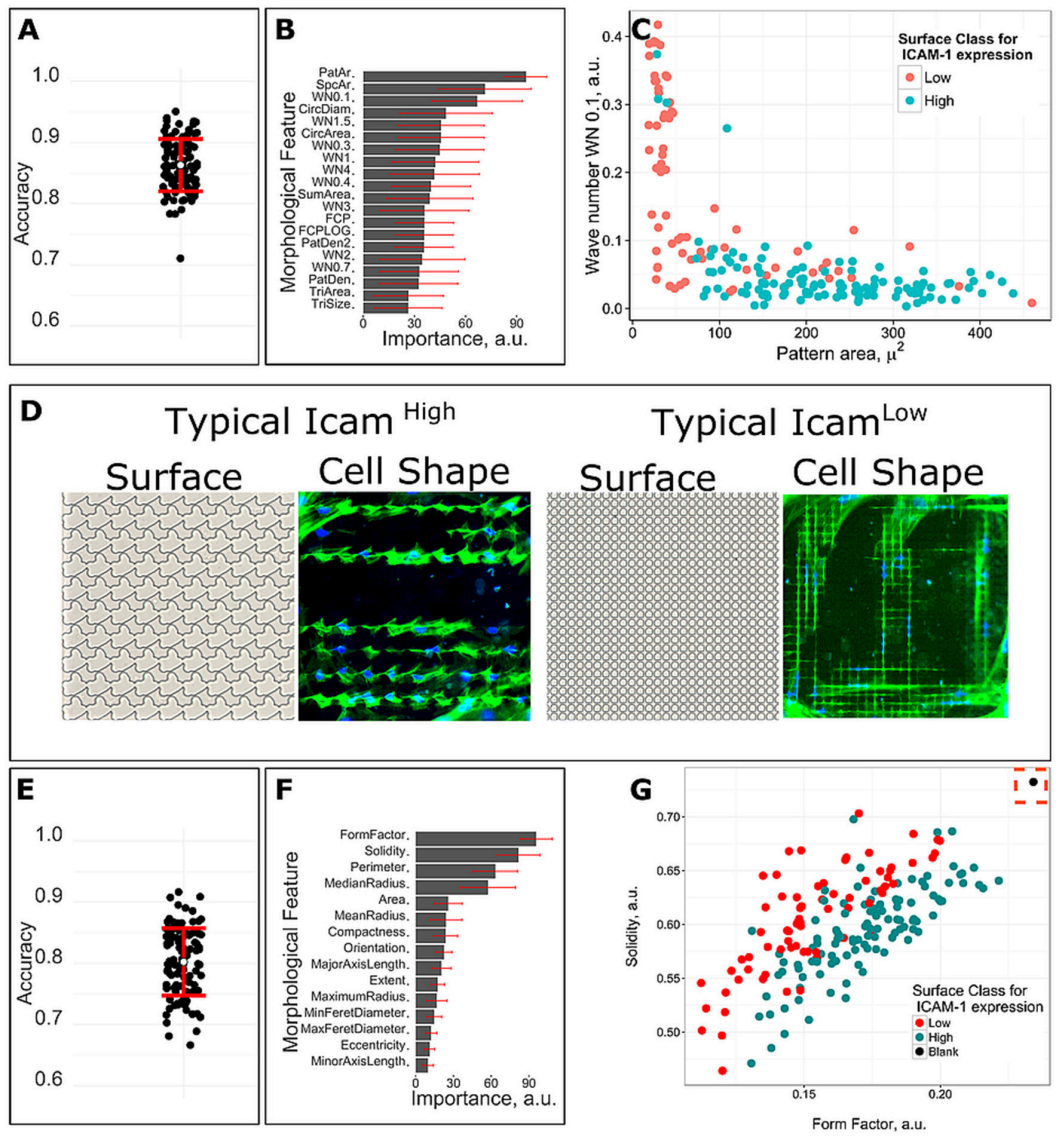
Correlations between surface design, cell shape and ICAM-1 expression. A machine learning model was trained with 10 fold cross-validation by employing random forest classification algorithm. Seventy-two low scoring units and one hundred twelve high-scoring units with significantly different frequency of ICAM-1 positive cells were used for the analysis. **(A)** The accuracy of 100 predicted models based on surface design parameters. **(B)** Feature mean and standard deviation of importance for predicting surface class for ICAM-1 level based on surface design parameters. **(C)** Surfaces with different level of ICAM-1 can be distinguished based on surfaces design parameters that were predicted as the most important by the model. **(D)** Typical cell morphologies and topography structure of ICAM-1^Low^ and ICAM-1^High^ surfaces. Green corresponds to actin and blue for nuclei. **(E)** The accuracy of 100 predicted models based on cell shape parameters. **(F)** Feature mean and standard deviation of cell shape parameters importance for predicting surface class for ICAM-1 level. **(G)** Surfaces with the different level of ICAM-1 can be distinguished based on cell shape parameters that were predicted as the most important by the model. Black dot is a control, cells on flat surfaces.

To evaluate the correlation between ICAM-1 expression and surface design parameters, we trained a machine learning model with 10-fold cross-validation by employing random forest (RF) classification, a pattern-recognition, computational algorithm that is able to unravel non-linear relationships in data. We used RF and binary classification to identify the surface design parameters that are unique to the 112 ICAM-1^High^ and 72 ICAM-1^Low^ surfaces. This was done by binary classification, which means that we had 2 different classes with ICAM-1^High^ or ICAM-1^Low^ surfaces and found similar design properties within those groups. We analyzed design parameters of seventy-two low scoring units and one hundred twelve high-scoring units that had a significant difference in frequency of ICAM-1 positive cells. The accuracy of the obtained model was assessed on a held-out data set, which was randomly selected from 1/3 of the original data, and not used for model training. To ensure robustness of the model, we performed model training 100 times with random splitting of the data into training and testing data sets. The average accuracy of the 100 simulations validated on the held-out data set was 86%, which means that from 100 surfaces, the algorithm was able to accurately predict expression of ICAM-1 on 86 of them (Figure 6A). In addition, we also quantified the importance of surface design parameters for separation of defined classes according to pattern recognition algorithm. The parameters that correlate to ICAM-1 expression based on average importance across all simulations (Figure 6B) include pattern area, space area, and the WN0.1 (Wave Number 0.1, which describes both density of structures and their size) (Figure 6C). A typical ICAM-1^High^ surface has pillars with the area in the range between 100 and 400 μm^2^ with moderate spacing between them (Figure 6D), while the typical ICAM-1^Low^ surface has densely located patterns with the area typically lower than 100 μm^2^ (Figure 6D).

Having observed that ICAM-1^High^ and I-CAM-1^Low^ surfaces resulted in different cell morphologies, we wished to obtain a model describing the relationship between cell shape and ICAM-1 expression, using the same machine-learning approach. The average accuracy of 100 simulations was 80% (Figure 6E). We found that the cell shape parameters Solidity, Form Factor, Perimeter, and Median Radius were the most important to separate the 2 classes in 100 simulations (Figure 6F), with Form Factor as the most important feature. Cells on flat surfaces were large and spread and thus had a large Form Factor value which is shown as a black dot in Figure 6G. At the same time, both positive and negative ICAM-1 cells were stretched elongated cells, but a typical ICAM-1 negative cell was thinner than ICAM-1 positive cells which also means more eccentric (Figure 6D).

### Validation of the screening results on enlarged surfaces

**Figure 7:**
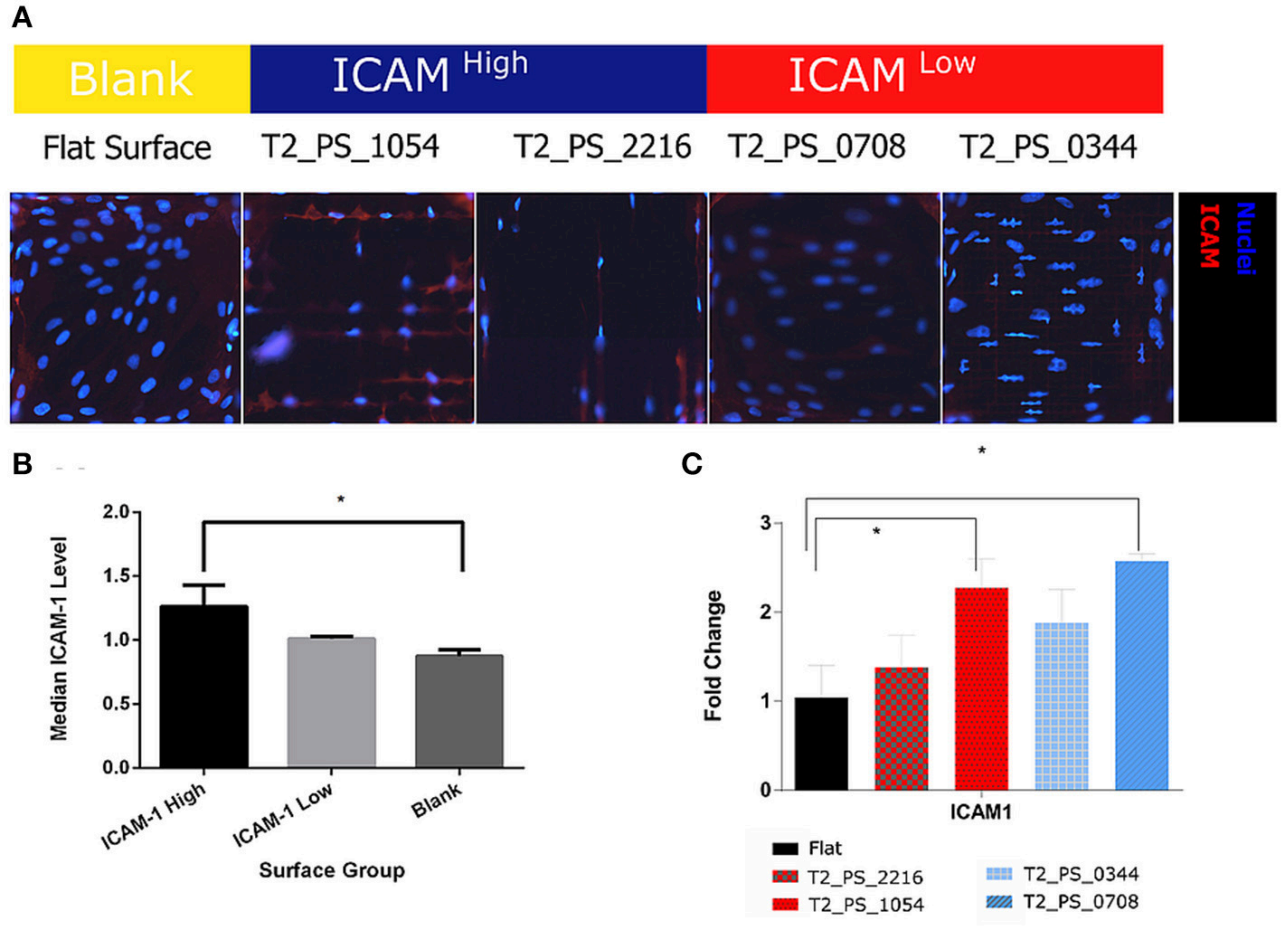
Validation of ICAM-1 staining. TSCs cells were grown on polystyrene topographical surfaces in basic media for 48 h. **(A)** Fluorescent images of ICAM-1 staining in TSCs, ICAM-1 is red, and nuclei are blue. **(B)** Median ICAM-1 level, quantified by FACS, results from hits topographies are pooled. **(C)** Expression of ICAM-1 on hits topographies measured by qPCR. *Indicates significant difference with a *p* < 0.05.

To validate results of our screening, we selected four topographies that belong to either ICAM-1^High^ or ICAM-1^Low^ (Figure 5C, Supplementary Figure 3) and have different cell shapes (Supplementary Figure 4), then fabricated enlarged surfaces (12 mm in diameter each). We cultured TSCs on one TopoChip for 48 h in basic medium without stimulation with TNF-α and LT-α1β2. TSCs were also cultured on these larger surfaces for 48 h without TNF-α and LT-α1β2. Cells were stained with DAPI to identify nuclei and antibodies to display ICAM-1 level. Similar to the screening data, the ICAM-1 expression levels on the larger surfaces were also different on ICAM-1^High^ and ICAM-1^Low^ topographies (Figure 7A). Using flow cytometry, we found a significant difference in ICAM-1 levels between TSCs on the flat control surface and the two ICAM-1^High^ surfaces (pooled medians) (Figure 7B).

To investigate whether upregulated ICAM-1 expression in TSCs on selected topographies correlates to co-expression of other FRC markers, we evaluated the expression of some FRC-specific genes using qPCR analysis. On ICAM-1^High^ surface and ICAM-1^Low^ surface ICAM-1 expression was significantly different with more than 2 – fold change in comparison with cells on the flat surface (Figure 7C). Interestingly, ICAM-1 gene expression level on the ICAM^High^ surface was the lowest of the three surfaces despite having the highest ICAM-1 expression at the protein level. None of the other markers of the FRC phenotype that we evaluated, such as VCAM-1, IL7, or CCL5, were differentially expressed in cells cultured on ICAM-1^High^ or ICAM-1^Low^ surfaces (data not shown).

**Figure 8:**
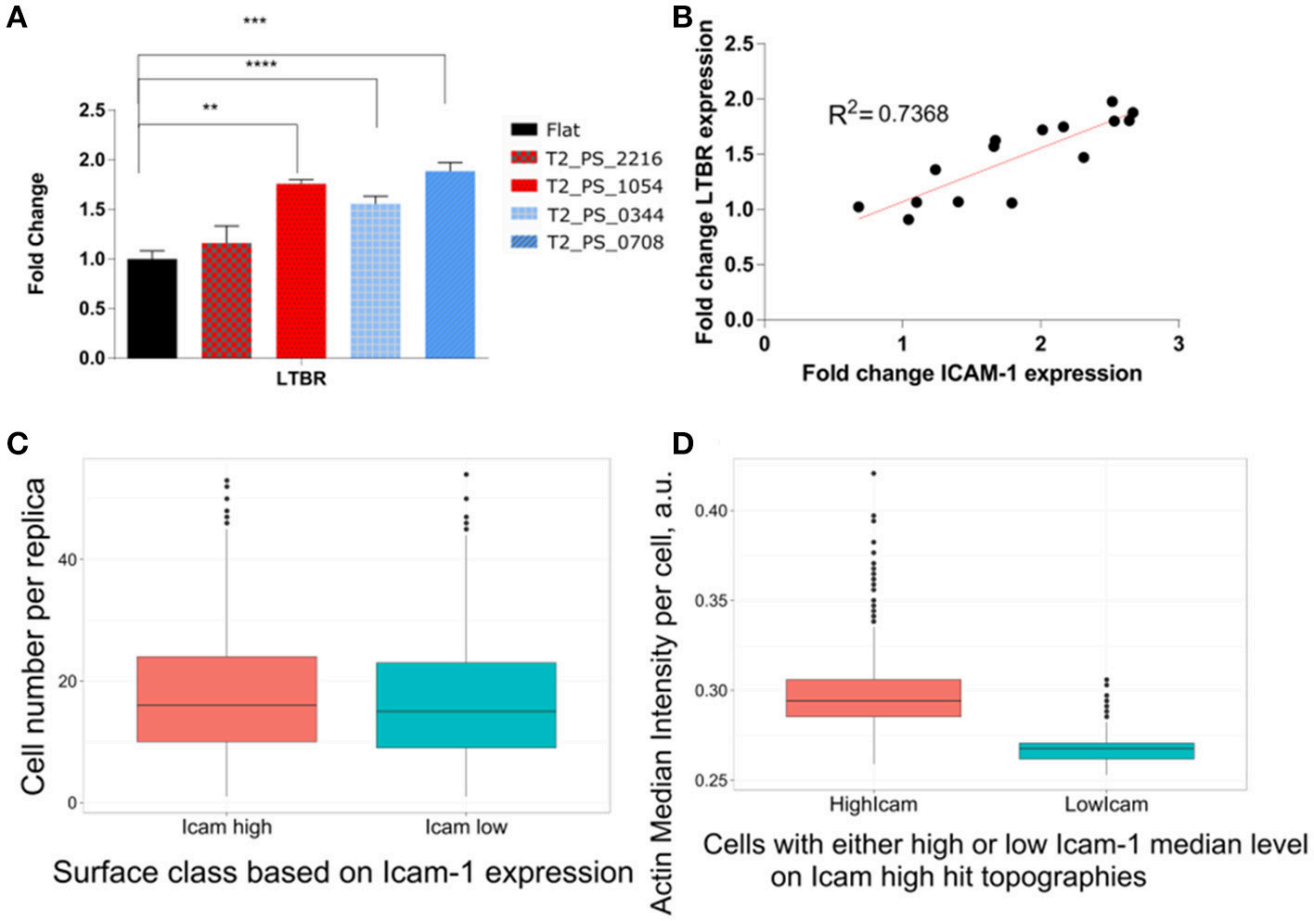
Expression of FRC phenotypical markers. TSCs cells were cultured on polystyrene topographical surfaces in basic media for 48 h. **(A)** Expression of LTBR on selected topographies measured by qPCR. **(B)** Correlation between fold change in LTBR and ICAM-1. **(C)** Differences in Cell number on ICAM-1^High^ and ICAM-1^Low^ topographies. TSCs cells were cultured on 8 Topochips in basic media for 48 h. A number of cells were quantified based on nuclei staining. **(D)** Actin intensity comparison between cells with high and low median ICAM-1 level on ICAM-1^High^ topographies. TSCs cells were cultured on 8 Topochips in basic media for 48 h. Actin Intensity was measured in images of cells stained with fluorescently labeled phalloidin. *Indicates significant difference with a *p* < 0.05. **Indicates significant difference with a *p* < 0.01. ***Indicates significant difference with a *p* < 0.001. ****Indicates significant difference with a *p* < 0.0001.

However, we did observe an increase of LTBR gene expression on all four surfaces containing topography. The possibility of modulation of LTBR by the topography by our knowledge was not reported before, and certainly needed further investigation (Figure 8A). Moreover, expression of ICAM-1 and LTBR highly correlated on selected topographies with *R*^2^ equal to 0.73 (Figure 8B).

We were able to validate results of the screening by immunofluorescent imaging, however, we did not observe consistent upregulation of FRC specific phenotypical markers.

## Discussion and conclusion

The objective of this study was to investigate whether TSCs are mechanoresponsive, and topography alone is sufficient to induce an FRC phenotype. We used ICAM-1 as readout because it is strongly upregulated during differentiation from stromal progenitors to FRC, making it a sensitive marker (Bénézech et al., 2012). ICAM-1 expression strongly increases in response to TNF-α and LT-α1β2, and ICAM-1 is an adhesion molecule known to be involved in mechanotransduction (Lessey-Morillon et al., 1950; Marjoram et al., 2014). Although ICAM-1 is an intercellular adhesion molecule (Rothlein et al., 1986), it has been reported as responsible for cell-cell interaction between lymphocytes and endothelial cells (Luscinskas et al., 1991). Moreover, it is known to be transcriptionally activated by the mechano-sensitive transcription factor egr-1 (Zhang et al., 2013). However, before our study, there has been no evidence that intercellular adhesion molecules can be manipulated by topography. Our investigation shows that ICAM-1 expression can be manipulated by topographical cues.

Differences in the number of ICAM-1–positive cells per unit may be explained by differential adhesion of ICAM-1–positive cells or by the proliferative advantage of ICAM-1–positive cells on selected topographies. Subpopulations of TSCs have an intrinsic expression of ICAM-1 (Ame-Thomas et al., 2007). Importantly, we did not observe surfaces where all cells were ICAM-1–positive in all replicas. However, the difference between the median cell numbers on ICAM-1^Low^ topographies and ICAM-1^High^ topographies is negligible (16 vs. 17 cells, respectively), which makes differential growth or adhesion advantages unlikely (Figure 8C). Similarly, topographies do strongly influence migratory behavior of cells, as has been reported for other cell types (Park et al., 2016). However, our TopoChip contains walls between units that make it unlikely that subpopulations of ICAM-1–positive cells migrated to hit topographies. Furthermore, we could predict accurately the class of surfaces (ICAM-1^High^ or ICAM-1^Low^) from the screening based on surface design and cell shape data. Moreover, the differences between selected hits were clearly visible on immunostained images (Supplementary Figure 4), indicating that topography induced ICAM-1 expression in TSCs.

The different levels of ICAM-1 in TSCs on ICAM-1^High^ surfaces might be related to cell cycle, as expression heterogeneity is well known for primary cell lines (Sivasubramaniyan et al., 2012; Gothard et al., 2014). Whether this applies for TSCs here requires further investigation. Interestingly, ICAM-1 intensity directly correlated to actin intensity (Figure 8D). Actin is the main transducer of mechanical signals to the cell (Gaspar and Tapon, 2014); if the actin network is less filamentous, it also provides less signal for ICAM-1 upregulation. It is known that actin directly binds to adhesion molecules such as ICAM-1 via ezrin, radixin, and moesin complexes (Lévesque and Simmons, 1999; Neisch and Fehon, 2011). Therefore, it is very likely that the expression of ICAM-1 is regulated through a mechanotransduction pathway involving actin. The effect of mechanical loading on the level of ICAM-1 has been described: an increase in interstitial flow can upregulate ICAM-1 in lymphatic endothelial cells (Swartz and Lund, 2012). This finding suggests that upregulation of ICAM-1 is a part of the global cell response to mechanical cues. For future work, it will be interesting to use cytoskeleton-disrupting agents to see how they affect ICAM-1 expression on the topographies.

In our validation studies with FACS, we saw small differences between selected topographies and flat surfaces which can be explained by first upregulation of ICAM-1 is not very high in comparison with TNF-α and LT-α1β2 and that this is bulk measurement. For such studies with heterogeneous cell population method that allows single-cell analysis, such as single cell qPCR, should be preferred.

Our findings of elongated cell shape correlating with I-CAM1^High^ surfaces are consistent with TSCs *in vivo*. Stromal cells in tonsils experience mechanical stress during the acute phase of inflammation when many lymphocytes enter the tonsil and subsequently expand the organ. For lymph nodes, it was shown that the total number of stromal cells does not increase, rather individual stromal cells stretch (Fletcher et al., 2015) during the early stages of organ expansion. Our elongated, ICAM-1–positive TSCs phenotypically resemble stretched cells.

Although a clear correlation was found between ICAM-1 expression, cell shape, and surface design, the cells did not adopt a mature FRC phenotype. We hypothesize that mechanotransduction can support but not fully trigger FRC differentiation.

TSCs respond very strongly to our library of topographies. We noted that TSCs are able to penetrate between dense pillars, and seem to do this more efficiently than BM-MSCs (Figure 3B). Different cell types behave differently on micropatterned topography, for example, Lecric et al. showed that when cultured together, fibroblasts tend to grow on ridges, while epithelial cells grow inside the grooves (Leclerc et al., 2013). Additionally, F. Badique et al. reported experiments on nuclear deformation on topographies with 3 osteosarcoma cell lines and demonstrated that cytoskeletal organization plays a major role in the cells' response to the microtopographies (Badique et al., 2013). It appears that TSCs more tightly adhere on topographical surfaces, and this may mimic their behavior in tonsils, where they have to create a very compact cellular structure and adhere to many cell types simultaneously. The findings reported here open up many avenues for further investigations. For example, it will be interesting to examine how topography affects meshwork formation in lymph node stromal cells.

In conclusion, we demonstrate that TSCs respond to surface topography and ICAM-1 expression correlates to both cell shape and surface design. Our ICAM-1–inducing surfaces are a valuable starting point to investigate the role of mechanobiology in FRC functions and in ICAM-1 mechanoregulation.

## Author contributions

AV and SS performed screening and analyzed the data. FM, AM, ML, and AV performed differentiation studies, flow cytometry analysis and qRT-PCR. FH, NB, and YZ fabricated TopoChip and enlarged surfaces. BP, AC, DS, CvB, KT, and JdB provided reagents and critically reviewed the manuscript before the submission.

### Conflict of interest statement

CvB and JdB are co-founders of and have a financial interest in Materiomics b.v. The remaining authors declare that the research was conducted in the absence of any commercial or financial relationships that could be construed as a potential conflict of interest.

## Supplementary material

Supplementary Figure 1 TopoChip. A micro topography screening platform contains 2176 unique topographies. The topographical features were randomly selected from an *in silico* library of more than 150 million of topographies, which were designed from an algorithm that synthesized patterns based on simple geometric elements – circles, triangles, and rectangles.

Supplementary Figure 2 Cells isolation schematic representation. Stromal cells were obtained from human tonsils collected from children undergoing routine tonsillectomy, after informed consent was obtained. Tonsils were cut into pieces and flushed using syringe and needle. Cell suspension was treated with DNAse I and collagenase IV followed by centrifugation on a discontinuous Percoll gradient. Cells were initially allowed to adhere for 48 h followed by elimination of nonadherent cells and culture in RPMI 1640 supplemented with 10% selected fetal calf serum, and penicillin/streptomycin.

Supplementary Figure 3 ICAM-1 expression on replicas from the screening. TSC cells were cultured on 8 Topochips in basic media for 48 h. DNA (blue) was stained with DAPI, ICAM-1 (Red) was stained using a combination of primary against ICAM-1 and specific secondary antibodies.

Supplementary Figure 4 Cell shapes of selected topographies. TSC cells were cultured on 8 Topochips in Basic media for 48 h. Actin (green) was stained with phalloidin, DNA (blue) was stained with DAPI.

Supplementary Figure 5 Distribution of ICAM-1 expression among replicas. Every dot is a median ICAM-1 expression in a single cell, in yellow corresponding box plot is showing. The adaptive threshold value for ICAM-1 positive cells is shown as a red line.

Supplementary Figure 6 Comparison of BM-MSC and TSC shapes on flat polystyrene and titanium coated surfaces. BM-MSCs were cultured in basic media for 5 days on titanium-coated flat surfaces and 24 h on polystyrene flat surfaces. TSCs cells were cultured for 48 h in basic media on polystyrene topographies.
