# Supplementary figures and images for: Designed Surface Topographies Control ICAM-1 Expression in Tonsil-Derived Human Stromal Cells
Source: Front Bioeng Biotechnol. 2018 Jun 28;6:87. doi: 10.3389/fbioe.2018.00087 (PMC6031747; doi:10.3389/fbioe.2018.00087)

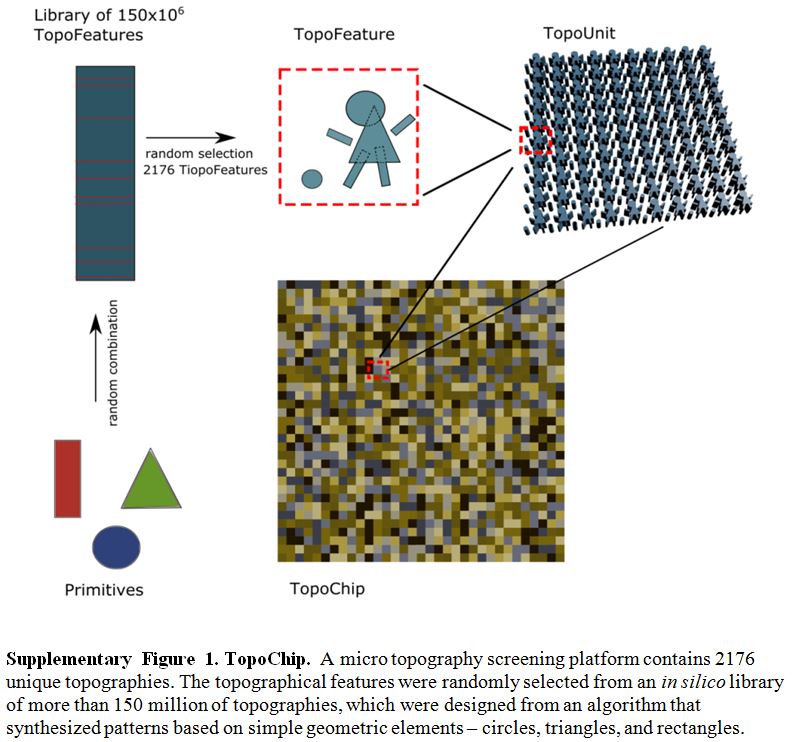

Supplement: Supplementary Figure 1 — TopoChip. A micro topography screening platform contains 2176 unique topographies. The topographical features were randomly selected from an in silico library of more than 150 million of topographies, which were designed from an algorithm that synthesized patterns based on simple geometric elements – circles, triangles, and rectangles. [file Image_1.JPEG]

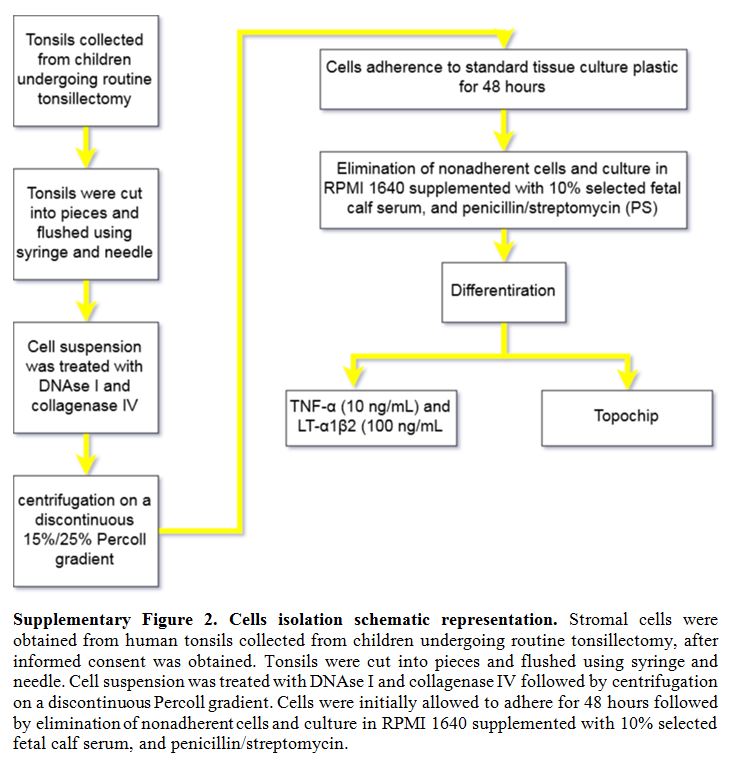

Supplement: Supplementary Figure 2 — Cells isolation schematic representation. Stromal cells were obtained from human tonsils collected from children undergoing routine tonsillectomy, after informed consent was obtained. Tonsils were cut into pieces and flushed using syringe and needle. Cell suspension was treated with DNAse I and collagenase IV followed by centrifugation on a discontinuous Percoll gradient. Cells were initially allowed to adhere for 48 h followed by elimination of nonadherent cells and culture in RPMI 1640 supplemented with 10% selected fetal calf serum, and penicillin/streptomycin. [file Image_2.JPEG]

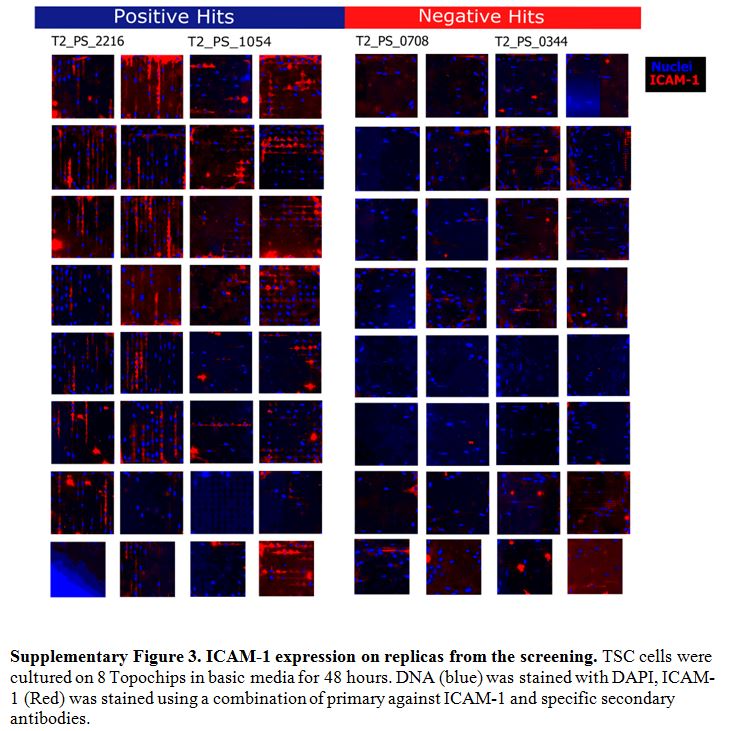

Supplement: Supplementary Figure 3 — ICAM-1 expression on replicas from the screening. TSC cells were cultured on 8 Topochips in basic media for 48 h. DNA (blue) was stained with DAPI, ICAM-1 (Red) was stained using a combination of primary against ICAM-1 and specific secondary antibodies. [file Image_3.JPEG]

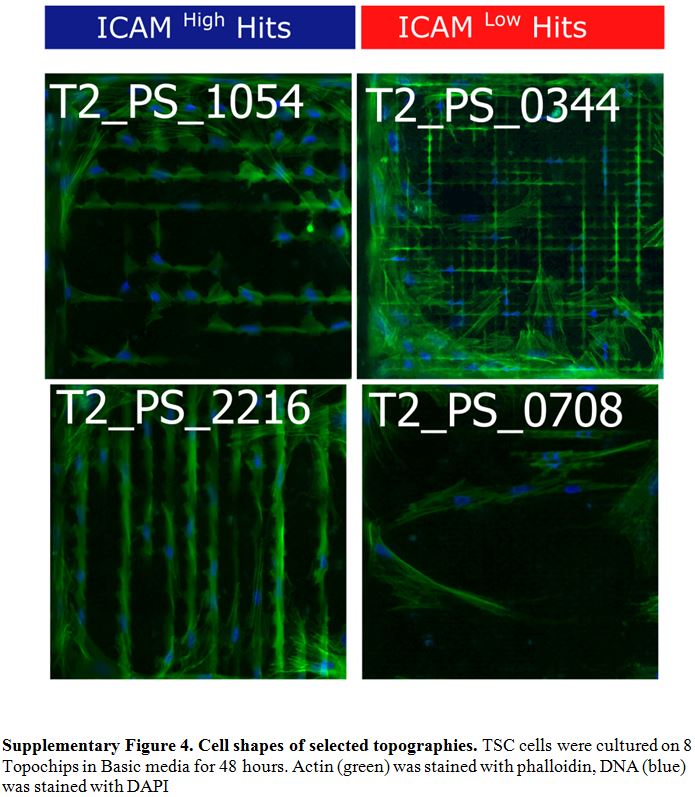

Supplement: Supplementary Figure 4 — Cell shapes of selected topographies. TSC cells were cultured on 8 Topochips in Basic media for 48 h. Actin (green) was stained with phalloidin, DNA (blue) was stained with DAPI. [file Image_4.JPEG]

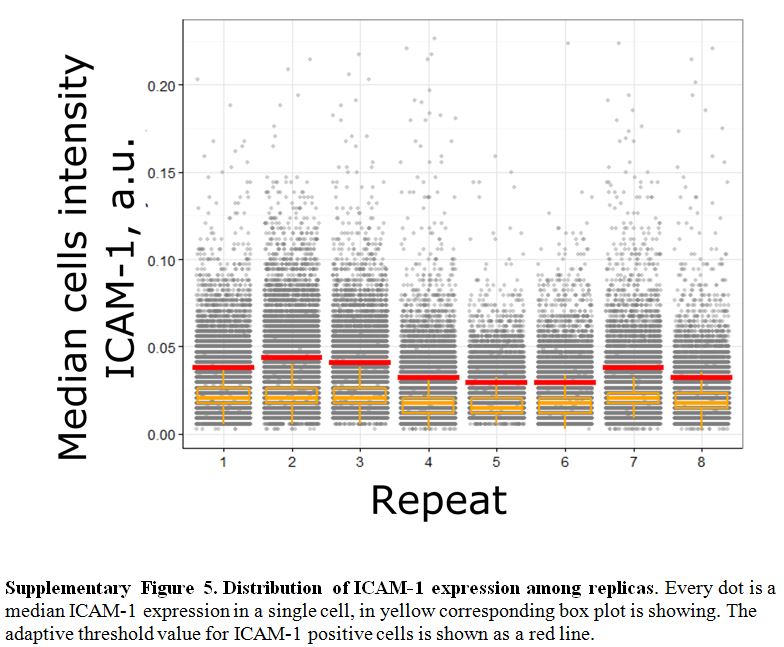

Supplement: Supplementary Figure 5 — Distribution of ICAM-1 expression among replicas. Every dot is a median ICAM-1 expression in a single cell, in yellow corresponding box plot is showing. The adaptive threshold value for ICAM-1 positive cells is shown as a red line. [file Image_5.JPEG]

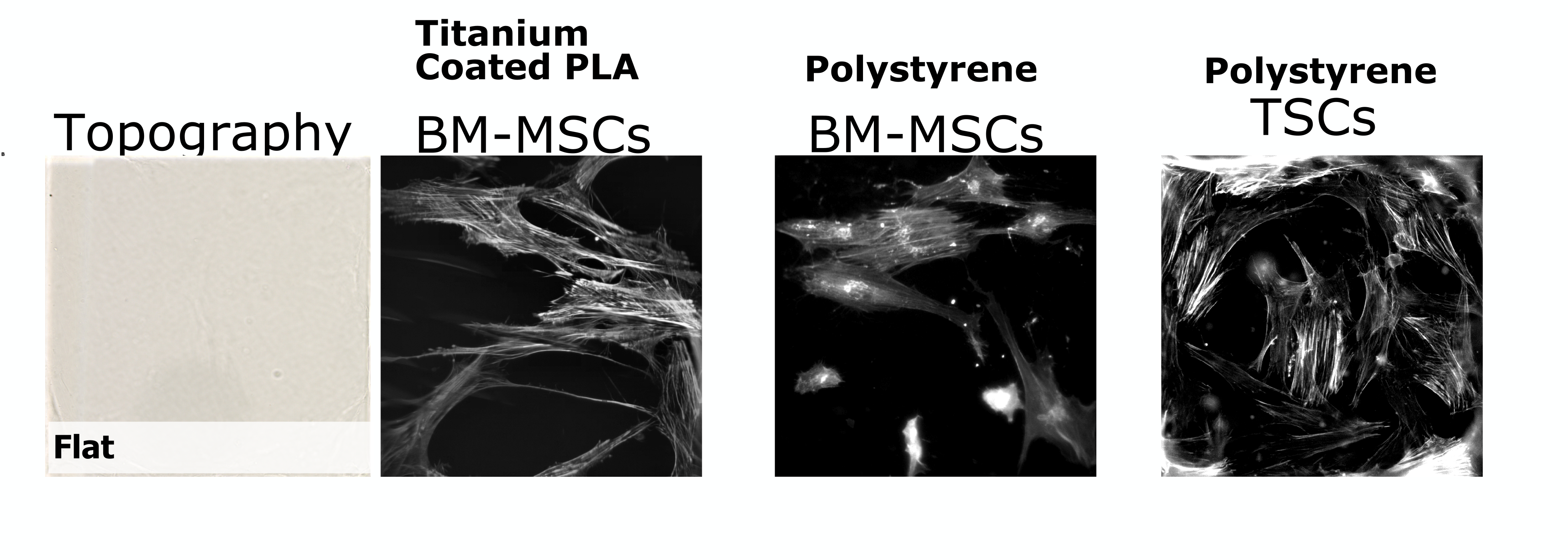

Supplement: Supplementary Figure 6 — Comparison of BM-MSC and TSC shapes on flat polystyrene and titanium coated surfaces. BM-MSCs were cultured in basic media for 5 days on titanium-coated flat surfaces and 24 h on polystyrene flat surfaces. TSCs cells were cultured for 48 h in basic media on polystyrene topographies. [file Image_6.PNG]
